# Cannabinoid Receptor 2 Agonist JWH-015 Inhibits Interleukin-1β-Induced Inflammation in Rheumatoid Arthritis Synovial Fibroblasts and in Adjuvant Induced Arthritis Rat via Glucocorticoid Receptor

**DOI:** 10.3389/fimmu.2019.01027

**Published:** 2019-05-08

**Authors:** Sabrina Fechtner, Anil K. Singh, Ila Srivastava, Christopher T. Szlenk, Tim R. Muench, Senthil Natesan, Salahuddin Ahmed

**Affiliations:** ^1^Department of Pharmaceutical Sciences, Washington State University College of Pharmacy and Pharmaceutical Sciences, Spokane, WA, United States; ^2^Preclinical COE, ETHICON, Medical Device Business Services, Inc., DePuy Synthes, Somerville, NJ, United States; ^3^Division of Rheumatology, University of Washington School of Medicine, Seattle, WA, United States

**Keywords:** fibroblasts, endocannabinoids, bone degradation, inflammation, antinociception

## Abstract

Management of pain in the treatment of rheumatoid arthritis (RA) is a priority that is not fully addressed by the conventional therapies. In the present study, we evaluated the efficacy of cannabinoid receptor 2 (CB2) agonist JWH-015 using RA synovial fibroblasts (RASFs) obtained from patients diagnosed with RA and in a rat adjuvant-induced arthritis (AIA) model of RA. Pretreatment of human RASFs with JWH-015 (10–20 μM) markedly inhibited the ability of pro-inflammatory cytokine interleukin-1β (IL-1β) to induce production of IL-6 and IL-8 and cellular expression of inflammatory cyclooxygenase-2 (COX-2). JWH-015 was effective in reducing IL-1β-induced phosphorylation of TAK1 (Thr^184/187^) and JNK/SAPK in human RASFs. While the knockdown of CB2 in RASFs using siRNA method reduced IL-1β-induced inflammation, JWH-015 was still effective in eliciting its anti-inflammatory effects despite the absence of CB2, suggesting the role of non-canonical or an off-target receptor. Computational studies using molecular docking and molecular dynamics simulations showed that JWH-105 favorably binds to glucocorticoid receptor (GR) with the binding pose and interactions similar to its well-known ligand dexamethasone. Furthermore, knockdown of GR using siRNA abrogated JWH-015's ability to reduce IL-1β-induced IL-6 and IL-8 production. *In vivo*, administration of JWH-015 (5 mg/kg, daily i.p. for 7 days at the onset of arthritis) significantly ameliorated AIA in rats. Pain assessment studies using von Frey method showed a marked antinociception in AIA rats treated with JWH-015. In addition, JWH-015 treatment inhibited bone destruction as evident from micro-CT scanning and bone analysis on the harvested joints and modulated serum RANKL and OPG levels. Overall, our findings suggest that CB2 agonist JWH-015 elicits anti-inflammatory effects partly through GR. This compound could further be tested as an adjunct therapy for the management of pain and tissue destruction as a non-opioid for RA.

## Introduction

In 1990, cannabinoid receptor 1 (CB1) was identified as the receptor responsible for tetrahydrocannabinol carboxylic (THC) effects (1). Three years later cannabinoid receptor 2 (CB2) was identified and together these receptors comprise the endocannabinoid system (ECS). CB1 is mainly expressed in the central nervous system and is primarily responsible for the psychoactive effects of cannabinoids concomitant to the neuroprotective effects (2). CB2 is mainly expressed peripherally, with its highest expression on immune cells. It is thereby associated with the immune suppressive and anti-inflammatory effect of cannabinoids (2, 3). Since the discovery of these receptors, endogenous ligands of the ECS such as anandamide (AEA) and 2-arachidonoylglycerol (2-AG) have been identified, and their synthesis and metabolism have been characterized. In addition to the endogenous ligands, several exogenous ligands have been identified such as cannabidiol (CBD). Although these ligands activate the ECS, they lack specificity to one receptor or the other. Thus, as our understanding of the ECS grows, specific ligands of either CB1 or CB2 are needed to characterize each receptor separately.

To address this need, John W. Huffman synthesized several ligands termed JWH compounds. Each ligand has differing affinities to CB1 and CB2 that can be used to activate one specific receptor over the other (4, 5). Many JWH compounds are now commercially available and can be used to help distinguish the differential effects of CB1 and CB2 activation.

Rheumatoid arthritis (RA) is an autoimmune disease characterized by inflammation and joint degradation. RA synovial fibroblasts (RASFs) are considered to be the main perpetrators by responding to pro-inflammatory cytokines interleukin-1β (IL-1β) and tumor necrosis factor-α (TNF-α) by producing IL-6, IL-8, and prostaglandins that exacerbate inflammation. Current therapies are targeted mainly to alleviate symptoms (non-steroidal anti-inflammatory drugs, NSAIDs) and slow down disease progression (disease-modifying anti-rheumatic drugs, DMARDs). The main goal of RA therapy is to increase remission rate in patients and one way to do so is to provide pain relief. However, pain management therapies are some of the most needed and demanded. A recent survey of 1,004 RA patients in the U.S. showed that 80% of those patients still experience pain daily or multiple time a week. In addition, 74% of them wished their therapies worked better (6). Indeed, another study showed that disease activity score-28 (DAS-28) does not reflect a reduction in pain in RA patients on current treatment options (7).

Recent literature suggests that the ECS may reduce both pain and inflammation in RA (3, 8, 9). Both CB1 and CB2 expression have been characterized in RASFs, where CB2 is upregulated in synovial tissue and RASFs. Within the synovial fluid of RA patients, AEA and 2-AG are found in detectable levels; however, both are undetectable in healthy joints. However, the reason for CB2 upregulation and the effects of CB2-targeted therapies remains unknown. JWH-133 is a specific CB2 agonist which has over 200 times more affinity for CB2 than CB1. Interestingly, JWH-133 administration reduced osteoarthritis pain-related behavior in the monosodium iodate-induced OA rat model (10). A study done by Selvi et al., using non-specific agonist CP 55,940 observed IL-1β-induced IL-6 and IL-8 production was inhibited with this agonist, however cytokine levels were not changed using CB1 and CB2 antagonists suggesting the presence of another anti-inflammatory receptor (11).

Therefore, the present study was carried out to evaluate the efficacy of CB2 selective agonist JWH-015 in human RASFs and *in vivo* using a rat model of RA. Upon further analysis, we identified that JWH-015 utilizes glucocorticoid receptor to produce anti-inflammatory affects.

## Materials and Methods

### Chemicals and Reagents

TRAF6, p-TAK^Thr184/187^, p-IRAK4^Thr345/Ser346^, IRAK4, p-P38, P38, p-JNK, JNK, p-ERK, ERK, GR, and NF-κBp65 antibodies were purchased from Cell Signaling Technologies (Danvers, MA) with respective catalog numbers 8028S, 90C7, D6D7, 4363, 4511S, 8690S, 9251S,9252T, 4370S, 4695S, 12041T, D14E12. p-TAK^Ser439^ was obtained from Abcam (Cat EPR2863). β-Actin and Lamin B antibodies were purchased from Santa Cruz Biotechnology (Santa Cruz, CA;, sc-47778, sc-6217). β-tubulin was purchased Sigma (St. Louis, MO cat# T8328). All antibodies were diluted in 5% BSA/TBS-T according to manufactures recommendation. JWH-015 was sourced from Tocris (Cat# 1341; ≥99% HPLC) and dissolved in DMSO at a stock concentration of 10 mM. For *in vivo* studies, JWH-015 was dissolved 3% DMSO/PBS.

### Culturing of Human RASFs

Human RASFs were isolated from patients diagnosed with RA according to the American College of Rheumatology (ACR) guidelines (7 female, 2 male, average age 50 ± 16.9 years). Briefly, de-identified human RA synovial tissues were obtained from Cooperative Human Tissue Network (CTHN; Columbus, OH) and National Disease Research Interchange (NDRI; Philadelphia, PA) according to an Institutional Review Board (IRB) approved protocol in compliance with the Helsinki Declaration. Synovial tissue was digested in Dipase, collagenase, and DNAase before being seeded in 72 cm^2^ flasks. Cells were grown in RPMI 1640 medium supplemented with 10% fetal bovine serum (FBS), 5000 U/ml penicillin, 5 mg/ml streptomycin, and 10 μg/ml gentamicin. Upon confluency (>85%) cells were passaged with brief trypsinization. All experiments were done using cells that were passed for additional 4 to 5 times to ensure enriched pure fibroblast population. For experimental purpose, we used RASFs between passages 5–10. All treatments were done in serum free media. All the experiments were performed on at least three or more cell lines established from different RA donors in this study.

### Treatment of RASFs

RASFs were seeded in 6-well plates and grown to >85% confluency. RASFs were pretreated with 10 or 20 μM of JWH-015 for 10 min prior to the addition of IL-1β (10 ng/mL). The duration of stimulation was for 30 min for signaling studies and/or 24 h to evaluate the production of IL-6, IL-8, and cyclooxygenase (COX) enzymes. Conditioned media was subjected to IL-6, IL-8, and PGE_2_ quantitation by ELISA, while whole cell extracts were used for the analysis of IL-1β signaling proteins like p-P38, p-JNK, p-ERK, and p-TAK-1Thr^184/187^using Western immunoblotting.

### Small-Interfering RNA (siRNA)

siRNA for CB2 [Catalog SASI_Hs01_00041077, SASI_Hs01_00041084, Sigma] and GR [SASI_Hs01_00188611, SASI_Hs01_00188614] were purchased from Sigma MISSION predesigned siRNA and RASFs were transfected as previously described (12). RASFs were transfected with 120 pmoles of negative (SIC001), CB2, or GR siRNA with Lipofectamine 2000 (Thermo Fisher Scientific) in Opti-MEM media for 8 h in 6 well format. Media was replenished with complete RPMI supplemented with 10% FBS and antibiotics next day. Forty-eight hours post transfection, RASFs were serum starved overnight prior to IL-1β simulation with or without JWH-015 for additional 24 h.

### Cell Fractionation

Cellular sub-fractionation to obtain nuclear and cytosolic fractions were performed as described previously (13). Briefly, RASFs were pretreated with 1 μM dexamethasone (Dex) 1 h or JWH-015 (20 μM) 10 min prior to IL-1β stimulation for 30 min. After preparation of cytoplasmic extract, nuclear pellet were subjected to 2–3 times sonication in RIPA buffer to obtain complete nuclear extract. Cytoplasmic and nuclear lysates were quantitated using Bio-Rad DC method followed by 25 μg of each treatment sample were subjected to Western immunoblotting. β-Tubulin was used for evaluating purity of cytosolic fraction and Lamin B was used for the nuclear fraction.

### Western Immunoblotting

Whole cell extract was prepared using RIPA buffer (50 mM Tris pH 7.6, 150 mM CaCl, 1% Triton X-100, 1 mM EDTA, 1 mM DTT, 0.5% sodium deoxycholate, and 0.1% SDS) containing protease and phosphatase inhibitors (Roche Basel, Switzerland). Protein was measured using BioRad DC method (Bio-Rad, Hercules, CA). Equal amount of protein (25 μg) for each sample was loaded and separated on a 10% acrylamide gel and transferred onto PVDF membrane (EMD Millipore, Billerica, MA). Blots were then blocked in TBST containing 5% nonfat dry milk for 2 h prior to overnight incubation with respective primary antibody with dilution according to manufacturer. Protein bands were visualized using chemiluminescence and analyzed using Image Lab software (Bio Rad) for band intensity. Blots were probed with β-actin to ensure equal loading.

### qRT-PCR

Treated RASFs were collected in 1mL of TRIzol Reagent (ThermoFisher Scientific, cat 15596026). RNA was extracted using the company provided protocol. 400 ng of RNA was used to make cDNA using Superscript II cDNA kit (ThermoFisher, cat 11904018). SYBR Green quantitative real-time PCR was used for analysis of CB2 (Sigma KiCqStart™ Primer H_CNR2_1) and GR (Qiagen QuantiTect primer GRQT00020608) with GAPDH (Qiagen QuantiTect primer QT00079247) as a control. Quantification of the relative expression was done using the ΔΔCt method.

### Assay for IL-6 and IL-8, PGE_2_, RANKL, and OPG Production

The conditioned media was collected from 24-h IL-1β stimulated samples with or without JWH-015, spun down at 10,000 rpm for 10 min at 4°C to remove particulate matter, and collected in fresh Eppendorf tubes. The collected supernatants were analyzed for human IL-6 and IL-8 levels using colorimetric sandwich ELISA kits (R&D Systems, Minneapolis, MN) as per manufacturer's instructions. PGE_2_ was assayed using colorimetric ELISA kit from Cayman Chemical (Ann Arbor, MI Cat# 514010) according to manufacturer's instructions.

RANKL and OPG were purchased from Ray Biotech (Norcross, GA cat # ELM-TRANCE-1 and ELM-OPG-1). Minor modification was made to OPG assay where samples and standard were incubated overnight at 4°C with gentle shaking. The remaining steps were performed as per manufacturer's instructions.

### Rat Adjuvant-Induced Arthritis (AIA)

All animal studies were approved by the ethics committee of the Washington State University and conformed to the NIH Guide for the Care the Use of Laboratory Animals (8th edition, 2011). Rat adjuvant arthritis studied was performed using similar parameters as described previously (14, 15). Briefly, ~120g female Lewis rats were purchased from Envigo (East Millstone, NJ) and allowed to acclimate for 1 week prior to start in campus vivarium accredited by the American Association for Accreditation of Laboratory Animal Care. On day 0, rats were administered 300 μl of 5 mg/ml lyophilized *Mycobacterium butyricum* (Difco Laboratories, Detroit, MI, USA) in sterile mineral oil subcutaneously at the base of the tail. Clinical parameters measured included articular index (AI) and ankle circumferences (AC) using parameters referenced in Ahmed et al. (14) AI scores were recorded for each hind joint by a consistent observer blinded to the treatment regimen and then averaged for each animal. AI scores were based on a 0–4 scale where 0 = no swelling or erythema, 1 = slight swelling and/or erythema, 2 = low to moderate oedema, 3 = pronounced edema with limited joint usage and 4 = excess oedema with joint rigidity. AC were also measured by the same blinded observer and the change in ankle circumference was presented as delta (Δ) ankle circumference. The Δ ankle circumferences of both the hind ankles from each animal were averaged and “n” is represented as the number of animals used in each of the experimental groups. Treatment of Animals with JWH-015.

JWH-015 was brought into suspension in phosphate buffered saline (PBS) with 3% DMSO. JWH-015 was administered daily via intraperitoneal injection (5 mg/kg) starting on day 9 after arthritis induction when the first signs of joint inflammation and swelling are usually noted and continued until day 17. On day 17, animals were sacrificed for biochemical, cytokine, and serum analysis.

### Behavioral Assays

Von Frey testing was performed 60 min after JWH-015 administration. Rats were acclimatized to testing cases for 15 min prior to testing. The amount of force applied to elicit a response (paw withdrawal or vocalization) was measured in grams. Three measurements were taken per hind paw with 30 s intervals in-between measurements and the order of paw testing (left vs. right paw first) was counter-balanced in each test group. In most cases, AIA arthritis was asymmetric where one paw was highly inflamed compared to the other thus, baseline data was compared between naïve, AIA, and JWH-015 groups was transformed to a ratio of the rat's own baseline score at day 8 compared to day 17. Therefore, mechanical threshold = [mechanical threshold in more inflamed paw- other hind paw at day 17]/[mechanical threshold in more inflamed paw-other hind paw at day 8].

### Imaging Studies: μ-CT Scanning

Ankles were fixed in formalin for preservation. One day prior to scan, ankles were placed in 1X PBS to mimic physiological conditions. Ankles were imaged using Quantum GX micro-CT Imaging System (Perkin Elmer Waltham, MA) using in-built “High Resolution Scan Mode.” Images were acquired at 90 kV and the standard total acquisition time was 4 min producing a 144 mm voxel image.

Bone mineral analysis was performed by standardizing images to QRM-MicroCT-HA phantom (QRM Moehrendorf, Germany). Rat tarsus bone was oriented vertically for analysis.

### Histological Analysis of Joint

For histological evaluation of the synovial joint, joints were decalcified with 10% EDTA for 14 days before being embedded with paraffin. Five μm slices were cut sagittally through the center line of the joint. Sections from naïve, AIA alone, and AIA + JWH-015 groups were stained with hematoxylin and eosin. Slides were photographed at 10 × magnification using Leica DM2500 microscope and were then evaluated for the presence of infiltrates, angiogenesis, and bone destruction.

For an objective evaluation of synovitis and inflammation, slides were analyzed for the following parameters: polymorphonuclear inflammation, immune cell infiltration (lymphocytes, plasma cells, and macrophages), neovascularization, and fibrosis (Supplementary Table 1).

### Molecular Dynamics Simulations

The binding pose and molecular interactions of JWH-015 with GR predicted by the docking simulations were further investigated by a 100 ns long molecular dynamics (MD) simulation of the docked complex. Prior to MD simulation, the crystal structure of the glucocorticoid receptor (PDB ID 4UDD) was prepared using MOE (16). Mutations introduced during protein crystallization were changed back to their respective wild type residues for the mutations, N517D, V571M, F602S, and C638D. The native sequence was preserved, all the amino acids were assigned their appropriate protonation states at pH 7.0, and miscellaneous ligands, water, and lipid molecules were removed. As the glucocorticoid receptor is primarily found in the cytosol of the cell, therefore, the receptor-ligand complex was simulated in a cubicle box of water with periodic boundary conditions (17). Charges and atom types of JWH-015 were assigned using the CGenFF server (18). The protein and water molecules were modeled using the CHARMM36 force field and TIP3P water model, respectively (19). The CHARMM-GUI input generator was used to setup all the simulated systems (20, 21). All MD simulations were run using the GPU version of NAMD 2.12 and trajectory analysis was done using visual molecular dynamics (VMD) software (22, 23). The system was neutralized (total charge equal to zero) by adding sufficient K^+^ ions (5) to the solvated receptor-ligand complex. The particle mesh Ewald method was used to treat long range electrostatic interactions (24). The non-bonded interaction list was generated with a distance cutoff of 14 Å and updated heuristically and Lennard-Jones interactions were truncated at 12 Å. The simulation was run at a constant pressure (1 atm) and temperature of 310 K. The temperature was controlled by using Langevin temperature coupling with a friction coefficient of 1 ps^−1^ (25). The pressure was maintained using a Nose-Hoover Langevin-piston method with a piston period of 50 fs and a decay of 25 fs (26). Covalent bonds to hydrogen atoms were constrained by SHAKE algorithm (27). The 1 fs/step time step was used in equilibration runs and 2 fs/step was used in production runs.

### Statistical Analysis

Statistical analysis was performed using GraphPad Prism Software. Data was analyzed using one-way ANOVA followed by Tukey's test for multiple comparisons test to determine which groups are significantly different from each other. Figures 3A,B were analyzed using two-way ANOVA followed by multiple comparisons test due to determine the effects of siRNA. All tests assumed normal distribution where α = 0.05 was considered significant. *In vitro* experiments were done in at least three different RA cell lines derived from three different RA patients; and data from at least six different rats are presented for *in vivo* experiments. All data are presented at mean ± SEM where error bars represent SEM.

## Results

### JWH-015 Is Anti-inflammatory in Human RASFs

To begin, we tested the effect of JWH-015 on common RA inflammatory markers (IL-6, IL-8, and COX-2). Before beginning studies, we performed a viability assay like the one described previously (28) where we saw no toxicity at the highest tested dose (20 μM). Based on the viability assay and previous literature (29), we selected the 10 and 20 μM doses of JWH-015 for *in vitro* studies.

**Figure 1:**
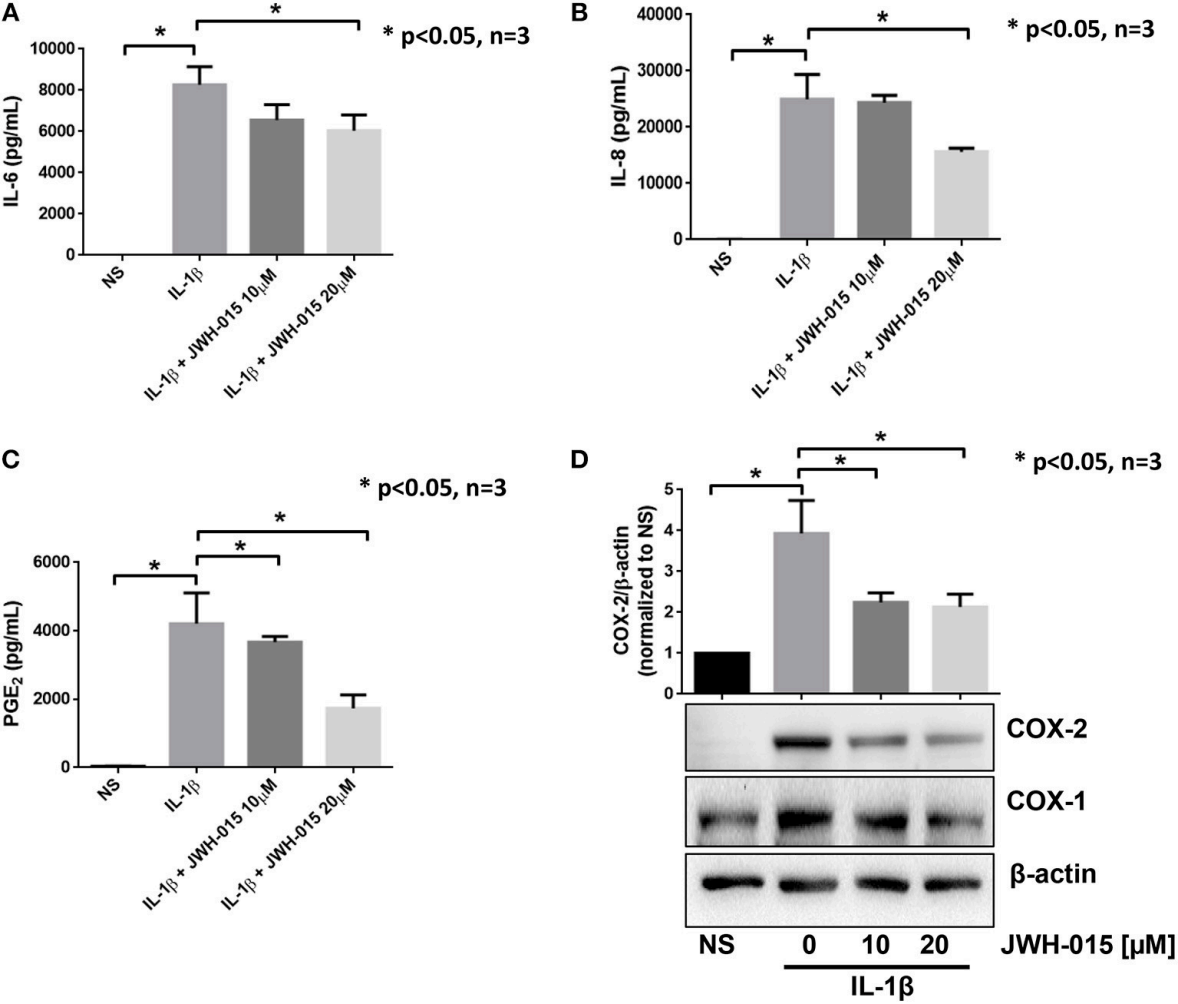
JWH-015 is anti-inflammatory in human RASFs. Upon confluency, RASFs were serum starved overnight prior to being pre-treated with JWH-015 (10 and 20 μM) for 10 min prior to the addition of IL-1β (10 ng/mL) for 24 h. Conditioned media was assayed for IL-6 **(A)**, IL-8 **(B)**, and PGE_2_ **(C)**, and **(D)** cell lysates were assayed for COX expression. JWH-015 at either dose did produce a response and was removed for clarity. Bars represent mean ± SEM of *p < 0.05, one-way ANOVA *n* = 3 where 3 different cell lines derived from 3 different RA patients was used. NS, non-stimulated.

RASFs were grown to <80% confluency before being serum starved overnight. JWH-015 was added 10 min prior to the addition to IL-1β (10 ng/mL) for 24 h. Evaluation of the conditioned media using ELISA assay showed a ~46% reduction of IL-6 and a ~50% reduction of IL-8 (Figures 1A,B). COX-2 expression was reduced significantly by ~40% correlating with a similar reduction of PGE_2_ production (Figures 1C,D).

### JWH-015 Inhibits IL-1β Induced Phosphorylation of TAK1

**Figure 2:**
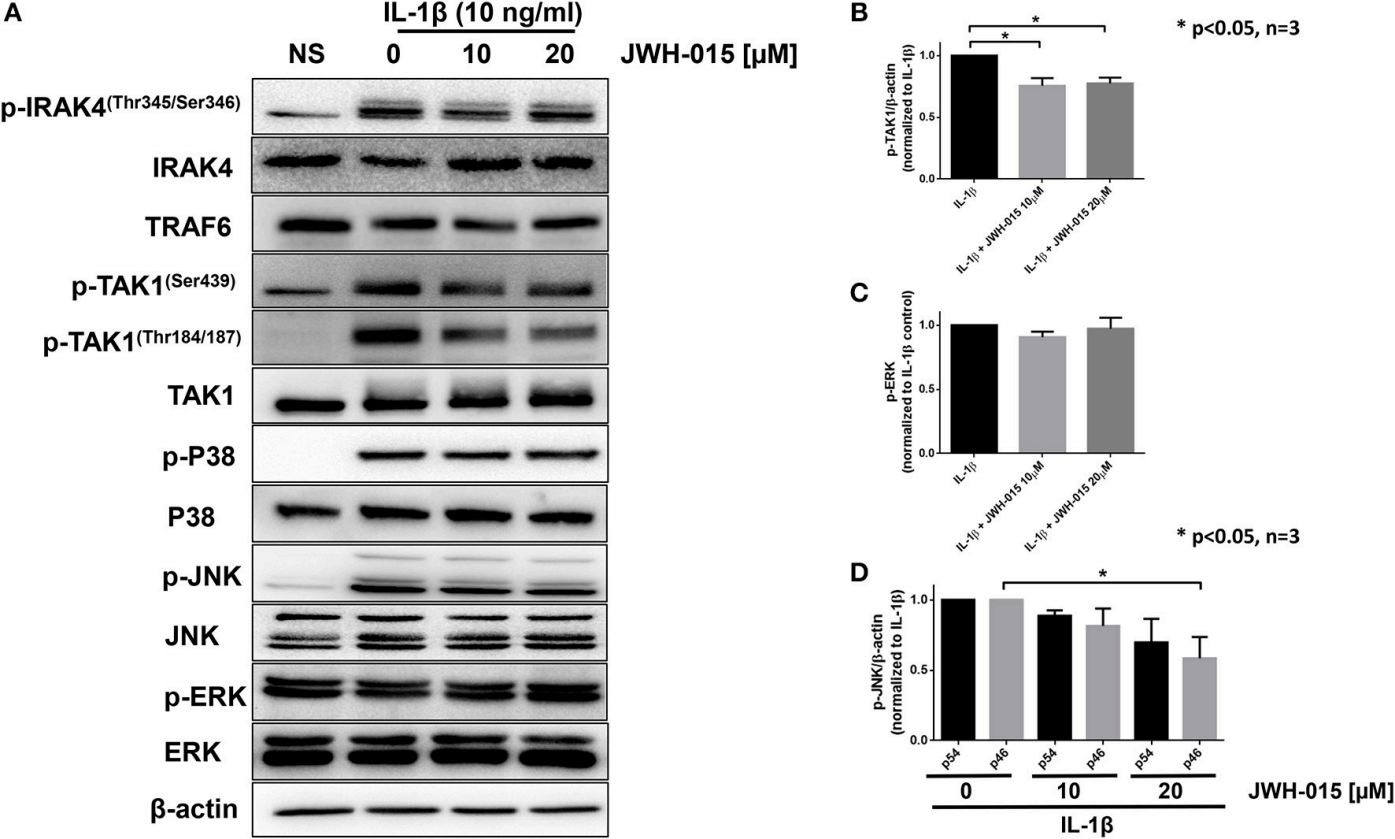
JWH-015 inhibits TAK1 activation. For signaling studies, RASFs were again pre-treated with JWH-015 (10 and 20 μM) for 10 min prior to the addition of IL-1β (10 ng/mL) for 30 min. **(A)** Cell lysates were collected and assayed for IL-1 signaling proteins using Western immunoblotting. Densitometric analysis of **(B)** pTAK-1^Thr184/187^, **(C)** p-ERK, and **(D)** p-JNK p54 and p46 is shown. JWH-015 alone at either dose did produce a response and was removed for clarity. *p < 0.05, one-way ANOVA *n* = 3 where 3 different cell lines derived from 3 different RA patients was used. NS, non-stimulated.

Because JWH-015 inhibited IL-1β-induced inflammation, we were interested in understanding the mechanism of action of JWH-015 in pretreated human RASFs. We examined the expression of key IL-1β proteins proximal to the IL-1 receptor (IRAK4/TRAF6/TAK1) to the downstream MAPKs (P38, JNK, ERK) in Figure 2A. Our Western blot results and densitometric analysis showed that JWH-015 inhibited the activation of p-TAK1^Thr184/187^, a site critical for its kinase activation and important to IL-1β signaling (30, 31). The inhibition of p-TAK1 resulted in a dose-dependent reduction in p-JNK activation, with no marked effect on p-P38 or p-ERK pathways (Figures 2B,C). Densitometric analysis showed that p-JNKp46 isoform was significantly inhibited at the highest dose (Figure 2D).

### JWH-015 Produces Anti-inflammatory Effects Independent of CB2

**Figure 3:**
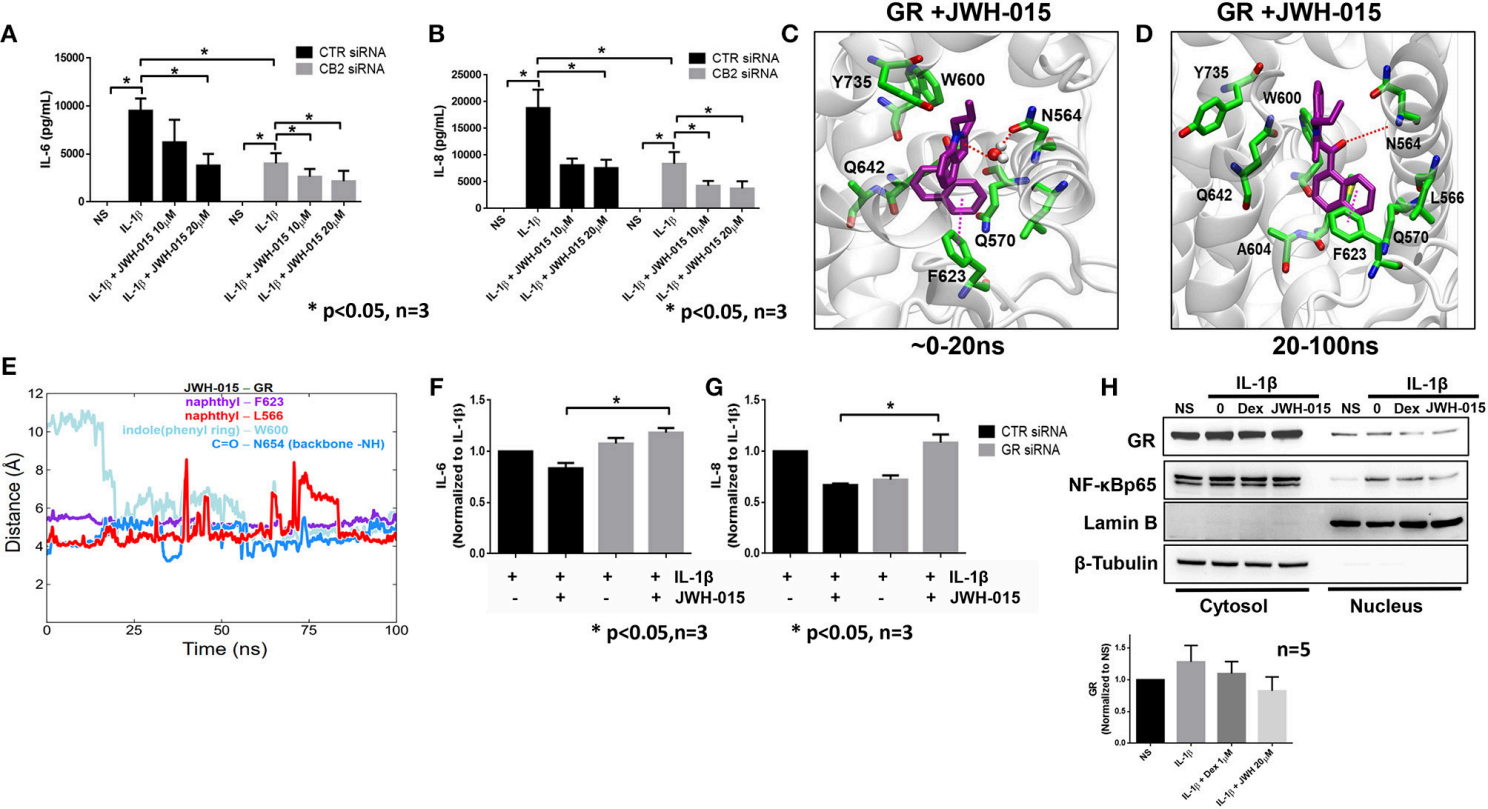
JWH-015 may utilize glucocorticoid receptor for anti-inflammatory action. CB2 was knocked down using 120 pM of targeted siRNA using Lipofectamine 2000 for 8 h. Media was replaced 24 h after transfection then serum starved overnight 48 h later. Next morning cells were stimulated with IL-1β (10 ng/ml) and JWH-015 (10 and 20 μM) for 24 h. Conditioned media was assayed for **(A)** IL-6 and **(B)** IL-8. JWH-015 was docked to the glucocorticoid receptor using MOE software. **(C)** A transient water-mediated electrostatic interaction was observed between the ligand and the sidechain carbonyl oxygen of N564 in GR; **(D)** The indole ring of the ligand flipped 180 degrees and enabling a lasting electrostatic interaction between carbonyl oxygen of the ligand and backbone –NH group of N564; **(E)** distances between the indole and naphthyl rings of the ligand and aromatic rings of W600 and F623 and side chain CH3 group of L566 indicating stable hydrophobic aryl-aryl and aryl-alkyl interactions. GR was knocked down using siRNA for 48 h prior to the addition of IL-1β ± JWH-015 (20 μM) for 24 h. Conditioned media was assayed for **(F)** IL-6 and **(G)** IL-8 production. **(H)** RASFs were pre-treated with Dex (1 μM) for 1 h or JWH-015 (20 μM) for 10 min before IL-1β for 30 min. Cytosolic and nuclear fractions were made and assayed for GR translocation. Densitometric analysis of GR nuclear localization in the presence of JWH-015 (20 μM) and Dex (1 μM). *p < 0.05, two-way ANOVA *n* = 3 where 3 different cell lines derived from 3 different RA patients was used. NS, non-stimulated.

Recent studies suggest that JWH-015 does not have high enough specificity to solely activate CB2 signaling (32, 33). Therefore, we wanted to examine if the anti-inflammatory action of JWH-015 observed in RASFs was through CB2 activation. Using small interfering RNA (siRNA), we knocked down CB2 expression and performed a similar experiment as shown in Figure 1 but with the addition of siRNA. CB2 knockdown was confirmed using Western immunoblotting and qRT-PCR (Supplementary Figures 1A,C) prior to data analysis. The knockdown of CB2 suppressed IL-1β-induced IL-6 and IL-8 production in human RASFs (Figures 3A,B). Interestingly, JWH-015 was still able to further inhibit IL-6 and IL-8 production even in the absence of CB2, suggesting JWH-015 may exploit non-canonical pathway independent of endocannabinoid receptors to elicit its anti-inflammatory effects (Figures 3A,B).

### JWH-015 Binds to Glucocorticoid Receptor

Next, we performed *in silico* molecular docking simulations to determine which receptors JWH-015 may bind to. We looked at receptors that are either known to bind endocannabinoid ligands or have anti-inflammatory effects in RASFs. One receptor which stood out to us was the glucocorticoid receptor (GR) because glucocorticoids have been used to treat RA since the 1950's (34). JWH-015 was docked to GR (PDB ID 4UDD) using MOE software program by induced-fit method allowing protein sidechain flexibility (16). Interestingly, the docked pose of JWH-015 within the GR binding site was found to be very similar to the bound structure of well-known GR ligand dexamethasone co-crystalized with the GR (Supplementary Figure 2A). The structurally fit binding pose along with the energetically favorable docking score (-8.0686 kcal/mol) accounting for interactions suggest JWH-015 may interact with GR.

The binding pose and molecular interactions of JWH-015 with GR predicted by the docking simulations were further investigated by a 100 ns long molecular dynamics (MD) simulation of the docked complex. The trajectory analysis (using VMD and in-house *tcl* scripts) revealed some interesting rearrangement (dynamics) of the ligand within the binding site (22). To begin, we observed a transient water-mediated electrostatic interaction between the indole ring nitrogen and the sidechain carbonyl oxygen of N564 that lasted for the first 20 ns (Figure 3C). After 20 ns into the simulation, the indole ring of JWH-015 flipped 180° (Figures 3C,D) from its initial orientation and engaged in lasting aryl-aryl interaction with W600. The napthyl ring was very stable in its docked orientation and was constantly surrounded by both F623 and L566 throughout the simulation (red and purple lines in Figure 3E). There is a strong and stable electrostatic interaction observed between the carbonyl oxygen of the ligand and backbone –NH group of N564 in the binding site. The distance between these two functional groups fluctuated between 3.5 and 5 Å throughout the simulation time (blue line Figure 3E) indicating strong electrostatic interactions.

We performed a similar simulation of JWH-015 docked to the CB2 receptor to compare the binding interactions (Supplementary Figures 2B,C). The trajectory analysis of CB2-JWH-015 complex revealed several hydrophobic interactions between the ligand and the binding site residues (Supplementary Figure 2B). The napthyl and indole rings of the ligand were well surrounded by several aromatic rings of F91, H95, F94, and F106. However, we did not observe any specific electrostatic interactions between the carbonyl oxygen of the ligand and the binding site residues nor any changes in the docked binding pose throughout the simulation time. Because hydrophobic interactions are inherently weaker than hydrogen bonds, MD simulations suggest that JWH-015 has an equal or stronger binding to affinity to GR than to CB2 (Supplementary Figure 2C).

To confirm *in vitro* simulation findings, we knocked down the GR receptor using siRNA for 48 h prior to the addition of IL-1β and JWH-015 for 24 h. Again, GR knockdown was confirmed using Western and qRT-PCR (Supplementary Figures 1B,D). The absence of GR completely abrogated the ability of JWH-015 to reduce IL-1β-induced IL-6 and IL-8 production (Figures 3F,G). Finally, we compared JWH-015 with dexamethasone (Dex) at inhibiting nuclear localization of GR. Indeed, JWH-015 showed a modest effect in inhibiting GR and NF-κBp65 nuclear localization, which suggests another possible mechanism of JWH-015's anti-inflammatory properties (Figure 3H).

### JWH-015 Is Anti-inflammatory in AIA Rat Model of Arthritis

**Figure 4:**
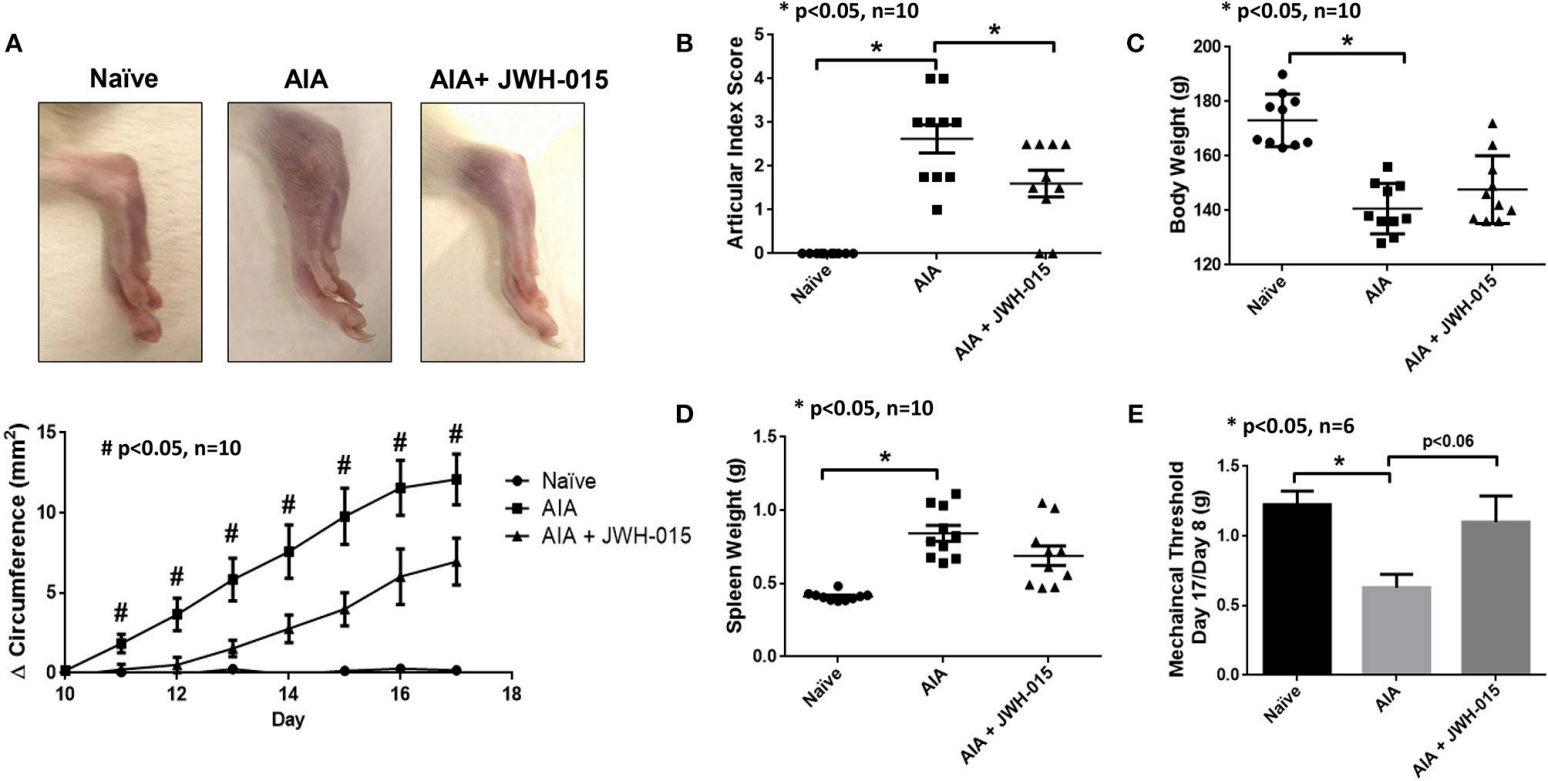
JWH-015 is anti-inflammatory in AIA arthritic rats. JWH-015 was given (5 mg/kg, ip) from day 8–17. Articular index scores were averaged for each animal **(B)** and Δ ankle circumferences **(A)** (normalized to day 0) values were averaged for each animal before group comparisons and final values represented as the mean of “n” number of animals per group. Mean ± SEM is shown for ankle articular index scores (range 0–4) and ankle Δ circumferences (in millimeters) determined on indicated days after adjuvant injection. **(D)** The mean ± SEM increases in body weight and spleen weight **(E)** measured on the final day of the experimentation. **(C)** Mechanical alloydina was measured with Von Frey test where a ratio of the rat's own baseline score at day 8 compared to day 17 was taken before averaged. The mean ± SEM in grams is presented here. *p < 0.05 one-way ANOVA *n* = 10 rats per group; *p < 0.05 one-way ANOVA *n* = 6 rats per group for Von Frey test. #*p* < 0.05 one-way ANOVA where all comparisons (naïve vs. AIA vs. AIA + JWH-015) are statistically significant *n* = 10 rats per group.

To confirm our *in vitro* findings, we tested the efficacy of JWH-015 *in vivo* using a rat AIA model of inflammatory RA. Rats were administered a daily intraperitoneal dose of JWH-015 (5 mg/kg) followed by AI scores and ankle circumferences measured starting day 9 (at the onset of arthritis) until termination (day 17). One of the few parameters JWH-015 administration was able to significantly reduce were ankle circumference and AI scores by 10.5% and 22.2%, respectively, by day 17 (Figures 4A,B) suggesting that JWH-015 is anti-inflammatory.

Von frey measurements were taken every other day beginning on day 8 at onset of inflammation. The amount of force applied in grams to elicit response (paw withdrawal or vocalization) was normalized to day 8 measurements. Rats began to show antinociceptive effects beginning on day 15 however, the effect was the strongest at day 17 (Figure 4E, *p* = 0.06). Cachexia is a common side effect of the AIA rat model therefore a gain in body weight shows signs of improvement overall improvement (35). In addition to cachexia, AIA also have enlarged spleens therefore a slight reduction of spleen weight at termination would suggest immunomodulatory effects (36). Although not statistically significant, JWH-015 was able to rescue body weight loss and reduce spleen enlargement in AIA rats at termination indicating reduction in overall inflammatory burden by JWH-015 without immunosuppression in this model (Figures 4C,D).

### JWH-015 Administration Prevents Bone Degradation in AIA Rats

**Figure 5:**
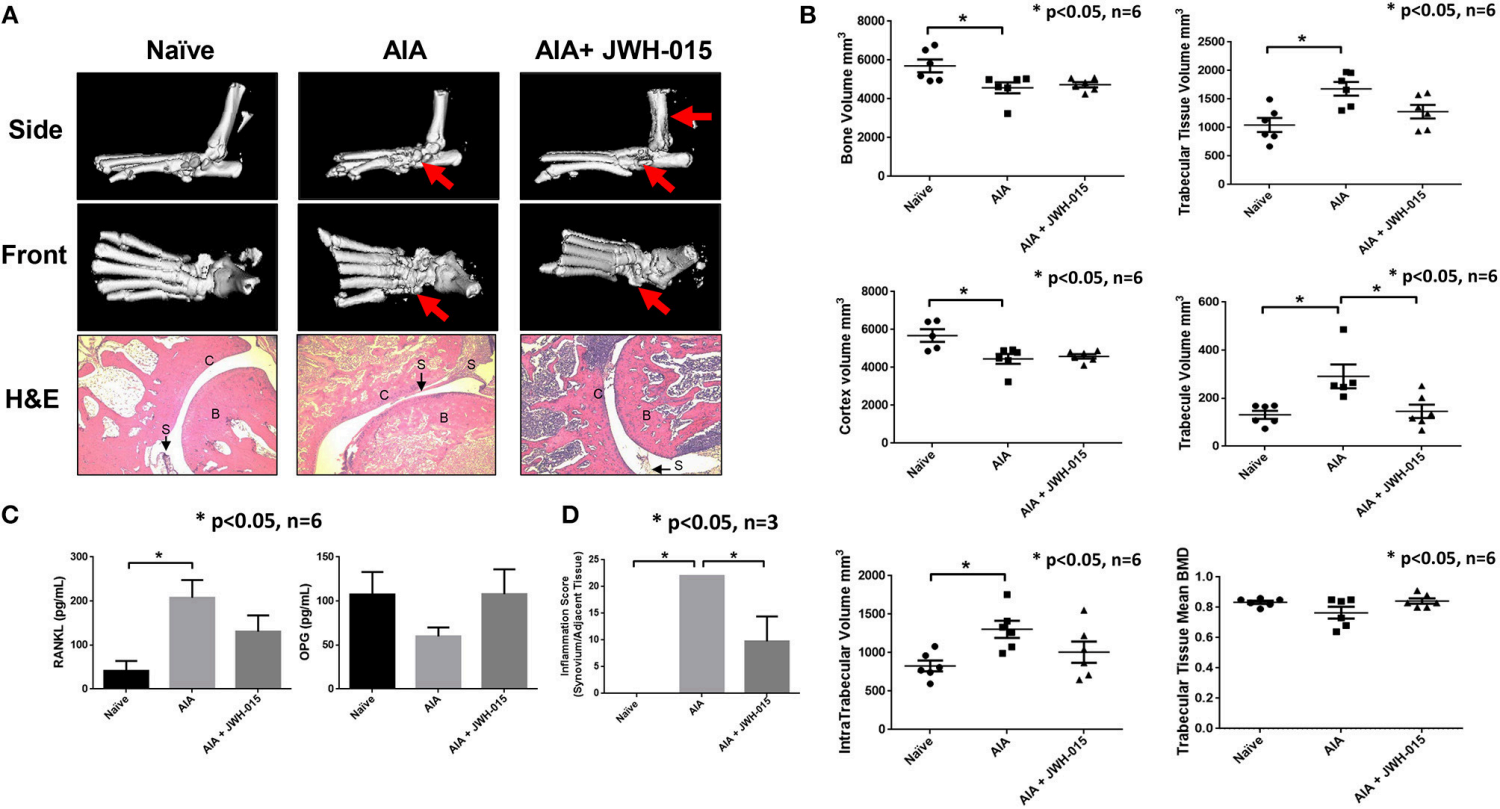
Less bone degradation is observed in animals given JWH-015. **(A)** μCT-imaging was done on the ankles using a “high resolution” scan mode after termination. Red arrows point to areas of apparent bone damage. Joints were then decalcified and 5 μm slices were stained for H&E. “B” marks bone; “C” marks cartilage; and “S” marks synovium. **(B)** Bone mineral analysis was performed on the rat tarsus bone calibrated to a phantom standard. **(C)** Rat serum was assayed for OPG and RANKL using ELISA method. Bars represent mean ± SEM *p < 0.05 *n* = 6 one-way ANOVA. **(D)** Histopathological analysis of H&E slides for synovial inflammation. Parameters and grading scale are described in detail in Supplementary Tables 1, 2. Scores were averaged per group where bars represent mean ± SEM *p < 0.05 *n* = 3 representative animals per group; one-way ANOVA.

To better understand the effects of JWH-015 on bone remodeling, we performed μCT-imaging on the ankles using a “high resolution” scan mode. Interestingly, we noticed portions of the AIA ankles that were damaged with disease that were not as severely damaged as compared to rats given JWH-015 (Figure 5A). Histological analysis was done on the treated and untreated rat joints, in which AIA rats had clear signs of synovitis and considerable amounts of inflammatory cell infiltration and bone loss (Figure 5D). In comparison, JWH-015 animals had less inflammatory cell infiltration and cartilage erosion (Figure 5A; H&E). Histopathological analysis showed a significant reduction in the inflammation score which included a reduction in immune cell infiltrates (lymphocytes, plasma cells, and macrophages) Figure 5D. Because synovitis is associated with pain, this suggests why JWH-015 may have an analgesic effect.

Furthermore, bone mineral density analysis showed distinct changes between naïve and AIA rats where trabecular tissue increased while bone and cortex volume deceased with disease (Figure 5B). Among different parameters studied, JWH-015 tended to prevent the trabecular bone from increasing in volume as demonstrated by the statistically significant lower trabecular volume. Again, many of the JWH-015 rats had similar values for intra-trabecular volume to that of the naïve rats, suggesting bone protective effect.

To further confirm these μCT and histological observations, we looked at the serum levels of RANKL and OPG which are important in bone reformation (37). We observed a statistically significant increase serum levels of RANKL in AIA rats and interestingly JWH-015 treated rats had lowered levels of RANKL and concomitantly increased the serum levels of OPG (Figure 5C). This suggests that JWH-015 can simultaneously reduce inflammation and bone destruction in inflammatory arthritis.

## Discussion

The findings from the present study provide an evidence for the anti-inflammatory role of a CB2 agonist JWH-015 in regulating IL-1β activated inflammatory responses in human RASFs and a rat AIA model of human RA. More importantly, we have identified how JWH-015 does not utilize CB2 for its anti-inflammatory actions, rather we demonstrated that JWH-015 has the capability of interacting with the GR receptor. Interestingly, the site of GR that JWH-015 binds is also exploited by dexamethasone, which may explain the observed inhibitory action triggered by inflammatory pathways. Based on our results revealing how JWH-015 may suppress pain and inflammation by targeting GR receptor in human synovial fibroblasts and in experimental animals, these findings may have rapid clinical application where JWH-015 or structurally similar molecules could be used as an adjunct a non-opioid therapeutic option for the management of pain and inflammation in RA.

JWH-015 has been reported for numerous biological activities, including anti-obesity, pro-apoptotic in thymic atrophy, anti- cancer, anti-inflammatory, and antinociceptive (29, 32, 38–41). In our study, we observed JWH-015 has anti-inflammatory effects in human RASFs as well as in a rat AIA model of arthritis. In recognizing JWH-015 does not have the strongest specificity to CB2, we anticipated a possibility of JWH-015 utilizing another receptor to produce anti-inflammatory effects. Our results from *in silico* molecular docking of JWH-015 to several potential receptors identified GR as a potential target. These findings were confirmed *in vitro* using siRNA approach to confirm that JWH-015 potentially rely on GR to elicit its anti-inflammatory actions.

Previous studies in RA have been done with other CB2 agonists. Gui et al. used the CB2 agonist HU-308 which has a Ki value of 22.7 nM to CB2 (9); Richardson et al used HU-210 which has a Ki of 0.52 nM at CB2 (8); and a more recent study used WIN 55,212-2 which has a Ki of 3.3 nM to CB2 (42). JWH-015 has a Ki value of 13.8 nM to CB2 and has been looked at in other cells that are involved in RA. In macrophages, JWH-015 was used to show that CB2 is not a chemoattractant receptor in primary murine macrophages and JWH-015 can inhibit chemokine-induced monocyte migration to inflammatory sites (43, 44). However, the underlying mechanism of its action and its effect on RA pathogenesis remains elusive.

At the highest concentration tested (20 μM), JWH-015 was effective in inhibiting IL-6, IL-8, and COX-2 expression, which are prominent inflammatory products of IL-1β signaling in RASFs (14, 31, 45). Upon looking further at the IL-1β signaling, JWH-015 elicits anti-inflammatory effects by inhibiting activation of p-TAK1, which correlated with the inhibition of p-JNK, and p-ERK expression. Previous studies have shown a similar inhibition of p-ERK by HU-308 and HU-210 in RASFs, but without any detailed analysis (8, 9). Interestingly within JNK/SAPK, JWH-015 preferentially inhibited p-JNKp46 isoform, which has been shown by us to be critically involved in IL-1β signaling as it binds to the AP-1 binding site with higher affinity than other JNK isoforms (46).

Although JWH-015 is defined as a CB2 agonist, its selectivity to CB2 is low compared to other readily available agonists. In a recent study, Craft et al. showed JWH-015 to induce anti-nociceptive responses in CFA-induced inflammation in rats via both CB1 and CB2 activation (32). In the rat AIA model, we also observed some analgesic effects, which we hypothesize to be independent of CB2 receptor based on our *in vitro* findings. A recent study done by Soethoudt et al., characterized several CB2 agonists including JWH-015. Using a panel of 64 proteins associated with common side effects from CEREP. The authors reported JWH-015 has 7 off-target receptors. Within the panel, they reported that JWH-015 has 36% efficacy for GR (33). GR is of particular interest to our findings because glucocorticoids have been used as first-line of treatment for RA since 1955 (34). *In silico* molecular docking of JWH-015 to GR not only produced a favorable binding score, but the docking pose of JWH-015 exhibited a striking resemblance to the bound poses of known GR ligand dexamethasone in the experimentally determined X-ray crystal structures. Molecular dynamic simulations reveals JWH-015 also forms additional hydrogen bonds with GR compared to CB2 where only hydrophobic interactions are observed.

Glucocorticoids mainly elicit their anti-inflammatory effects by interfering with pro-inflammatory transcription factors AP-1 or NF-κB from transcribing pro-inflammatory mediators, but other mechanisms of action for glucocorticoids are also described (34, 47). In our studies, JWH-015 inhibited GR translocation in a similar fashion to dexamethasone in RASFs. Although this result seems contradictory to GR's known mechanism of action, literature has shown that transrepression of GR is also important for its anti-inflammatory function where it can abrogate NF-κBp65 activation (48). Indeed, we observed JWH-015 was able to inhibit NF-κBp65 translocation to the nucleus. This suggests that JWH-015 mediated transrepression of GR into the nucleus may be responsible of JWH-015's anti-inflammatory action. In addition, the nuclear presence of GR can be misleading in that GR translocation does not necessarily produce anti-inflammatory effects. Pariante et al showed that IL-1α was able to enhance GR nuclear localization (49). As previously reported by our group, IL-1α has a similar function to IL-1β which suggests IL-1β can also enhance GR nuclear localization (13). Functional genomics studies are further warranted to validate GR response to extremal stimuli such as JWH-015. GR can also elicit its effects through non-genomic signaling which does not require translocation of GR into the nucleus. Non-genomic signaling has been reported to be important for the treatment of RA, but was not investigated in this study (50). Further studies are warranted to fully confirm that JWH-015 could mimic the functions of glucocorticoids without any adverse effects.

Among several limitations of long-term glucocorticoids use is progressive bone loss (47). Pharmacologically important, JWH-015 administration to AIA rats was able to ameliorate arthritis concomitant to preventing bone degradation. This could be strongly correlated with the significant reduction in serum RANKL and an increase in OPG in the treated rats around day 17 when arthritis peaks and inflammatory markers are highly expressed (36). While we observed histological and CT improvement in the limited window of JWH-015 treatment, extension of dose regimen and duration would have allowed us to validate if bone loss could be completely reverted to naïve levels in BMA analysis.

While the findings from this study are novel and clinically relevant, we acknowledge some limitations with our study. First, CB2 is also expressed in other cell types, including B cells, macrophages, and NK cells that have role in RA pathogenesis (3). Thus, characterizing the biological activity of JWH-015 in other cell types may help us understand the broader impact of JWH-015 or structurally similar molecules. Second, the study by Soethoudt et al., identified off target receptors that JWH-015 showed the binding affinity, including A3, 5-HT2A and 2B, and PPARγ.

In summary, JWH-015 exhibits anti-inflammatory action in human RASFs and in rat AIA model of RA. Further testing of JWH-015 in other models where its impact through GR could be validated may provide an opportunity to develop molecules on similar structure as an adjunct non-opioid analgesic and bone protective agents in inflammatory conditions such as RA.

## Ethics Statement

De-identified human RA synovial tissues were obtained from Cooperative Human Tissue Network (CTHN; Columbus, OH) and National Disease Research Interchange (NDRI; Philadelphia, PA) according to an Institutional Review Board (IRB) approved protocol in compliance with the Helsinki Declaration. The WSU Office of Research Assurances has determined that the study satisfies the criteria for Exempt Research at 45 CFR 46.101(b)(4)(IRB # 17249). All animal studies were approved by the IACUC committee of the Washington State University and conformed to the NIH Guide for the Care the Use of Laboratory Animals. The Approved protocol number is: 004957-006.

## Author Contributions

SF, AS, SN, and SA conceived and designed experiments. SF, AS, IS, and CS performed the experiments. SF, AS, SN, TM, and SA analyzed the data. SF, SN, and SA wrote the manuscript.

### Conflict of Interest Statement

TM is employed by the ETHICON. All other authors declare no competing interests.
