# Supplementary material for: Cannabinoid Receptor 2 Agonist JWH-015 Inhibits Interleukin-1β-Induced Inflammation in Rheumatoid Arthritis Synovial Fibroblasts and in Adjuvant Induced Arthritis Rat via Glucocorticoid Receptor
Source: Front Immunol. 2019 May 8;10:1027. doi: 10.3389/fimmu.2019.01027 (PMC6519139; doi:10.3389/fimmu.2019.01027)
Supplement: Supplementary file 1 [file Data_Sheet_1.docx]

Supplementary Material

# Supplementary Data

## Docking and MD simulation of JWH-015 bound to cannabinoid receptor (CB2)

The steps involved in protein preparation, docking and MD simulations were similar to those described earlier for the GR-JWH-015 complex, and only differences are discussed further. As there is no experimental structure available for JWH-015 bound to the CB2 receptor, docking simulation was undertaken to choose a likely binding pose for further MD simulation. The X-ray structure of the CB2 receptor (PDB ID 5ZTY) was downloaded from the Protein Data Bank (Li et al., 2019). The mutations introduced during crystallization (G78L, T127A, R242E, and G304E) were reverted to the original wild type version, and the bound T4-lysozyme was removed from the structure. The correct protonation states of all residues at pH 7.0 were assigned and other miscellaneous ligands, waters, and lipids were removed prior to simulation.

The ligand JWH-015 was docked to Phe87, Phe91, Phe94, and His95 as binding site residues. Mutations of these residues were shown to affect CP 55,940 (agonist) binding substantially to CB2/CB1 receptors (Li et al., 2019). As the CB2 receptor is a membrane bound GPCR, the receptor-ligand complex was first oriented in its native membrane environment made up of 1-palmitoyl-2-oleoyl-sn-glycero-3-phosphocholine (POPC) lipid bilayer using the OPM database (Lomize et al., 2012). The CHARMM-GUI membrane builder was used to set up the receptor-ligand complex (Wu et al., 2014). Protein and lipid were parameterized using the CHARMM36 force field, and again the ligand was modeled using the CGenFF parameters (Huang and MacKerell, 2013). The final simulation box dimensions were 75 x 75 x 99 Å with 64 and 65 lipids in the upper leaflet and lower leaflet of the bilayer respectively.

# Supplementary Figures and Tables

## Supplementary Figures


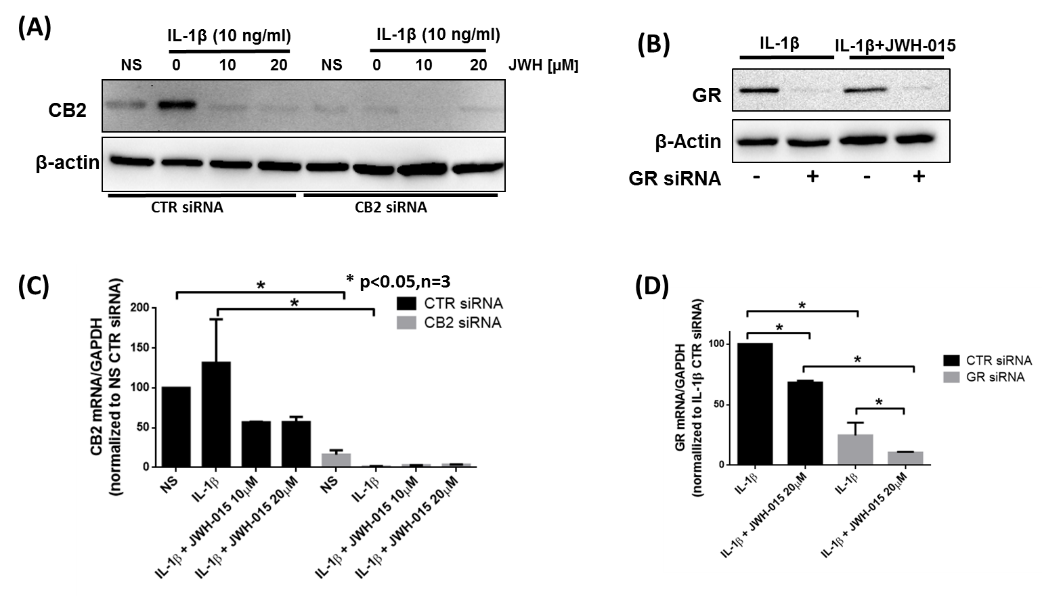


**Supplementary Figure 1.** *CB2 and GR Knockdown.*  RASFs were transfected with siRNA targeting CB2 (**A** and **C**) or GR (**B** and **D**) using Lipofectamine 2000 for 48 hours. Cells were pre-treated with JWH-015 for 10 minutes prior to IL-1β stimulation. For qRT-PCR, IL-1β stimulation was for 8 hours (**C** and **D**); and 24 hours (**A** and **B**) for Western immunoblotting.


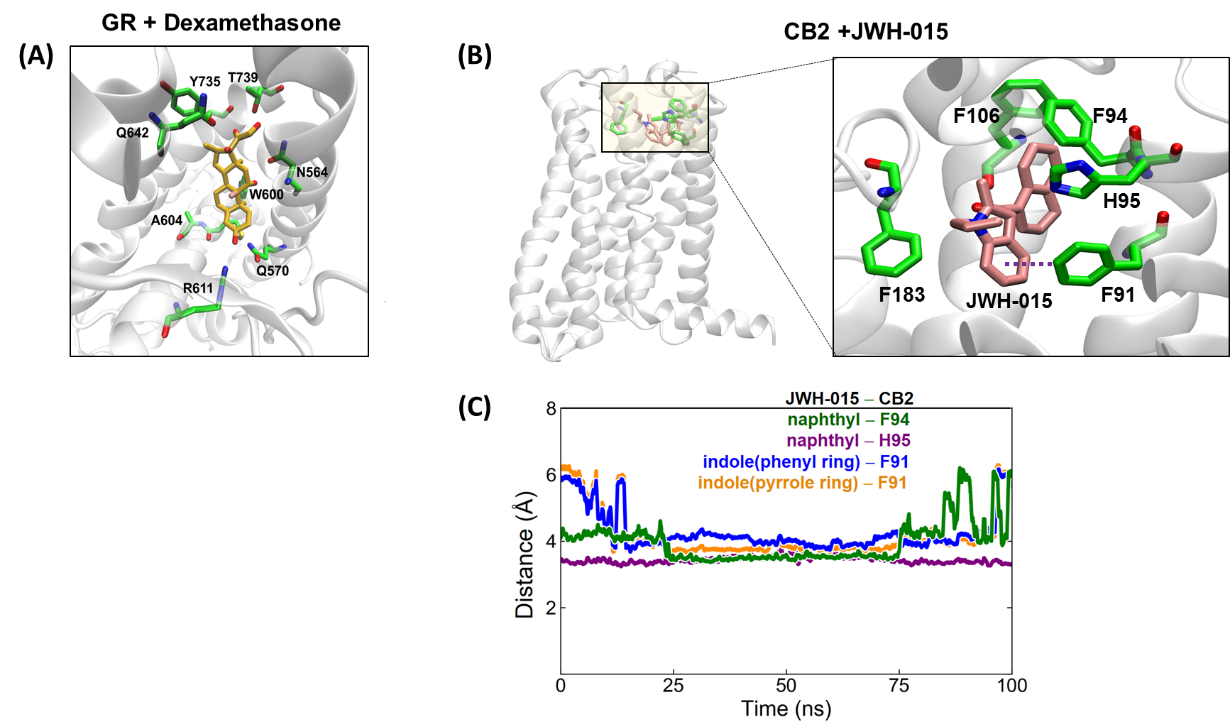


**Supplementary Figure 2.** *JWH-015 bound to CB2 receptor*. (**A**) The most favorable docking pose of of dexamethasone (yellow) a known ligand for the GR. (**B**) JWH-015 surrounded by various aromatic residues; (**C**) distances between the indole and napthyl rings of the ligand and the aromatic rings of the binding site residues.

## Supplementary Tables

| CELL TYPE/RESPONSE | 0 | 1 | 2 | 3 | 4 |
| --- | --- | --- | --- | --- | --- |
| Polymorphonuclear cells (PMNS) | 0 | Rare, 1-5/hpf | 6-10/hpf | Heavy infiltrate | Packed |
| Lymphocytes | 0 | Rare, 1-5/hpf | 6-10/hpf | Heavy infiltrate | Packed |
| Plasma cells | 0 | Rare, 1-5/hpf | 6-10/hpf | Heavy infiltrate | Packed |
| Macrophages | 0 | Rare, 1-5/hpf | 6-10/hpf | Heavy infiltrate | Packed |
| Giant cells | 0 | Rare, 1-2/hpf | 3-5/hpf | Numerous | Sheets |
| Necrosis | 0 | Minimal | Mild | Moderate | Severe |
| Neovascularization | 0 | Minimal capillary proliferation, focal, 1-3 buds | Groups of 4-7 capillaries with supporting fibroblastic structures | Broad band of capillaries with supporting structures | Extensive band of capillaries with supporting fibroblastic structures |
| Fibrosis | 0 | Narrow band | Moderately thick band | Thick band | Extensive band |
| Edema | none | minimal | mild | moderate | severe |
| Global inflammation severity score | none | minimal | mild | moderate | severe |
| Synovial lining cell layer grade | none | minimal | mild | moderate | severe |
| Cartilage surface change/erosion | normal | irregular, chondrocyte degeneration evident | clefts, limited-to-minimal articular cartilage loss | clefts extend to bone, focal-to-coalescing cartilage loss |  |
| Subarticular bone erosion/loss grade | none | minimal | mild | moderate | severe |
| Bone peripheral to joint loss | no loss | minimal | mild | moderate | severe |
| Evidence of new bone/bone remodeling | none | minimal | mild | moderate | marked |

**Supplementary Table 1.** *Inflammation of periarticular tissues.* Histopathological parameters and grading system used for assessing pathology in rat joint slides stained with H&E.

|  | Inflammation- synovium/adjacent tissue | | | | | | | | | | | | | | |
| --- | --- | --- | --- | --- | --- | --- | --- | --- | --- | --- | --- | --- | --- | --- | --- |
| Treatment group | Naïve | | | Non-treated-AIA | | | | | Treated-AIA | | | | | | |
| Joint | 1 | 2 | 3 | 1 | 2 | 3 | 4 | | 1 | 2 | | 3 | | 4 | |
| Inflammation  Polymorphonuclear | 0 | 0 | 0 | 4 | 4 | 4 | 4 | | 0 | 4 | | 1 | | 2 | |
| Lymphocytes | 0 | 0 | 0 | 1 | 1 | 1 | 1 | | 0 | 1 | | 0 | | 0 | |
| Plasma Cells | 0 | 0 | 0 | 0 | 0 | 0 | 0 | | 0 | 0 | | 0 | | 0 | |
| Macrophages | 0 | 0 | 0 | 4 | 4 | 4 | 4 | | 0 | 4 | | 1 | | 2 | |
| Giant Cells | 0 | 0 | 0 | 0 | 0 | 0 | 0 | | 0 | 0 | | 0 | | 0 | |
| Necrosis | 0 | 0 | 0 | 0 | 0 | 0 | 0 | | 0 | 0 | | 0 | | 0 | |
| SUB TOTAL (X2) | 0 | 0 | 0 | 18 | 18 | 18 | 18 | | 0 | 18 | | 4 | | 8 | |
| Neovascularization  (fibroplasia) | 0 | 0 | 0 | 2 | 2 | 2 | 2 | | 0 | 2 | | 1 | | 2 | |
| Fibrosis | 0 | 0 | 0 | 2 | 2 | 2 | 2 | | 0 | 2 | | 1 | | 1 | |
| SUB TOTAL | 0 | 0 | 0 | 4 | 4 | 4 | 4 | | 0 | 4 | | 2 | | 3 | |
| TOTAL | 0 | 0 | 0 | 22 | 22 | 22 | 22 | | 0 | 22 | | 6 | | 11 | |
| GROUP TOTAL | 0 | | | 88 | | | | | 39 | | | | | | |
| AVERAGE* | 0.0 | | | 22.0 | | | | | 9.8 | | | | | | |
|  | | | | | | | | | | | | | | | |
|  |  |  |  |  |  |  | |  |  | |  | |  | |  |
| Edema in soft tissues | 0 | 0 | 0 | 3 | 3 | 3 | | 3 | 0 | | 3 | | 1 | | 3 |
| Global inflammation severity score | 0 | 0 | 0 | 2 | 3 | 3 | | 3 | 0 | | 3 | | 1 | | 2 |
| Synovial lining cell  layer grade | 0 | 0 | 0 | 3 | 3 | 3 | | 3 | 1 | | 3 | | 1 | | 3 |
| Cartilage surface change/erosion | 0 | 0 | 0 | NA^§^ | 3 | 3 | | 3 | 1 | | 3 | | 1 | | NA^§^ |
| Subarticular bone erosion/loss grade | 0 | 0 | 0 | NA^§^ | 4 | 3 | | 3 | 1 | | 1 | | 0 | | NA^§^ |
| Bone peripheral to joint loss | 0 | 0 | 0 | 3 | 4 | 4 | | 4 | 0 | | 4 | | 1 | | 1 |
| Evidence of new bone/bone remodeling | 0 | 0 | 0 | 2 | 2 | 2 | | 2 | 1 | | 2 | | 1 | | 1 |

**Supplementary Table 2.** *Microscopic Evaluation Individual findings of ankle joint.* Detailed analysis of pathology of rat AIA joint slides stained with H&E. Four representative slides from diseased rats in case one slide was of poor quality and could not be assessed. However all slides sent were indeed scored. § The tissue section of the bone/joint was not near the center of the joint, the evaluation of the articular surface of interest in the tissue section was limited and not considered adequate for full evaluation.

# Supplementary References

Huang, J., and MacKerell, A.D., Jr. (2013). CHARMM36 all-atom additive protein force field: validation based on comparison to NMR data. *Journal of computational chemistry* 34(25)**,** 2135-2145. doi: 10.1002/jcc.23354.

Li, X., Hua, T., Vemuri, K., Ho, J.H., Wu, Y., Wu, L., et al. (2019). Crystal Structure of the Human Cannabinoid Receptor CB2. *Cell* 176(3)**,** 459-467.e413. doi: 10.1016/j.cell.2018.12.011.

Lomize, M.A., Pogozheva, I.D., Joo, H., Mosberg, H.I., and Lomize, A.L. (2012). OPM database and PPM web server: resources for positioning of proteins in membranes. *Nucleic acids research* 40(Database issue)**,** D370-D376. doi: 10.1093/nar/gkr703.

Wu, E.L., Cheng, X., Jo, S., Rui, H., Song, K.C., Dávila-Contreras, E.M., et al. (2014). CHARMM-GUI Membrane Builder toward realistic biological membrane simulations. *Journal of computational chemistry* 35(27)**,** 1997-2004. doi: 10.1002/jcc.23702.
